# Supplementary figures and images for: The APOBEC3B cytidine deaminase is an adenovirus restriction factor
Source: PLoS Pathog. 2023 Feb 6;19(2):e1011156. doi: 10.1371/journal.ppat.1011156 (PMC9934312; doi:10.1371/journal.ppat.1011156)

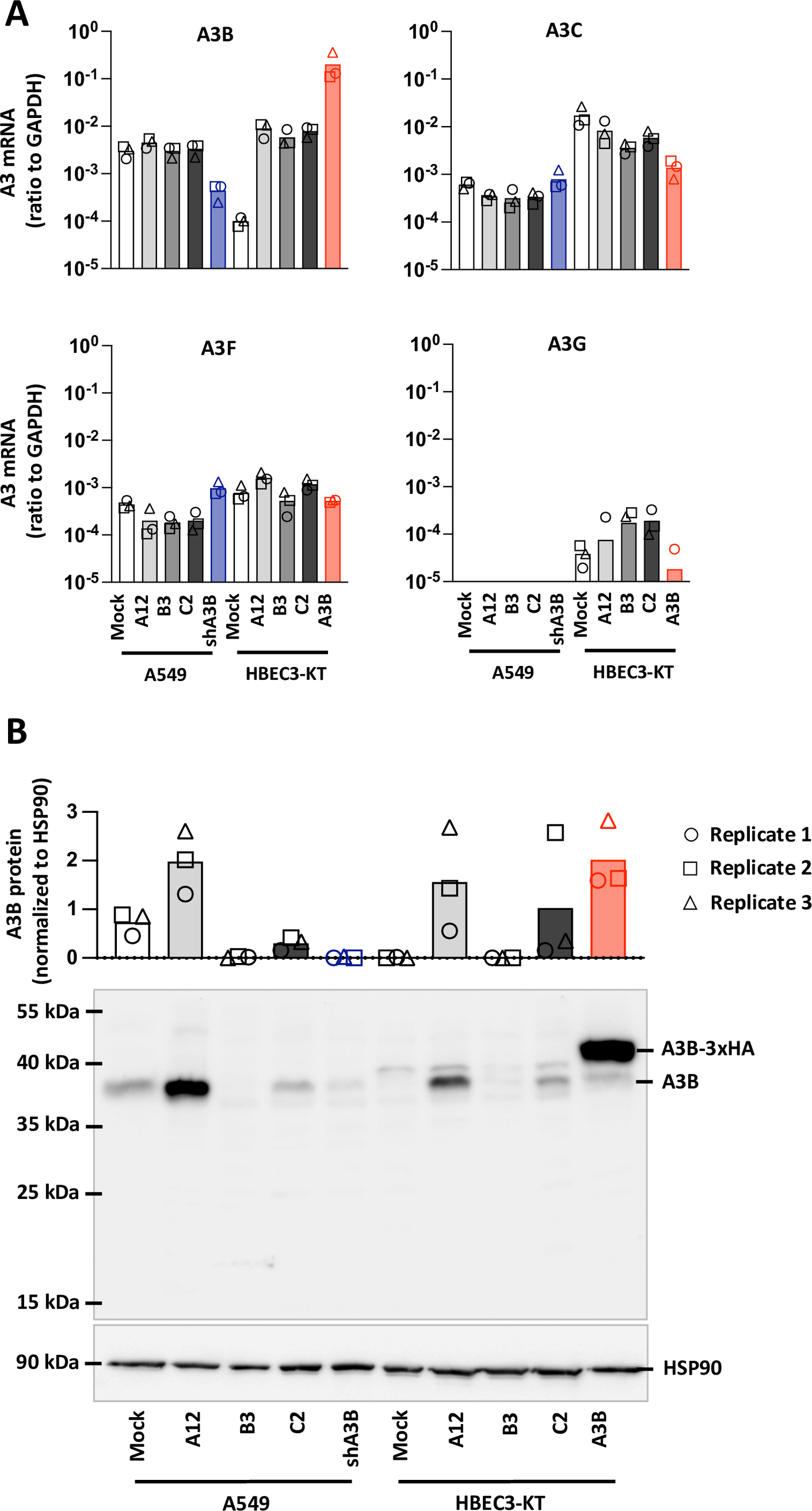

Supplement: S1 Fig — A549 cells were infected with HAdV-A12, -B3, -C2 or mock control at a MOI = 3 and analyzed 2 days post infection. HBEC3-KT were infected with HAdV-A12, -B3, -C2 or mock control at a MOI = 1 and analyzed 4 days post infection. A3s expression levels were also reported in uninfected A549 shA3B and HBEC3-KT A3B. (A) The A3 mRNAs were quantified by RT-qPCR and expressed relative the GAPDH. The A3A, A3D and A3H mRNAs were not detected. (B) The A3B protein levels were assessed by western blot using the 5210-87-13 mAb antibody, quantified by densitometry and normalized with HSP90. Panel B displays a full blot from 15kDa to 55 kDa. (TIFF) [file ppat.1011156.s001.tiff]

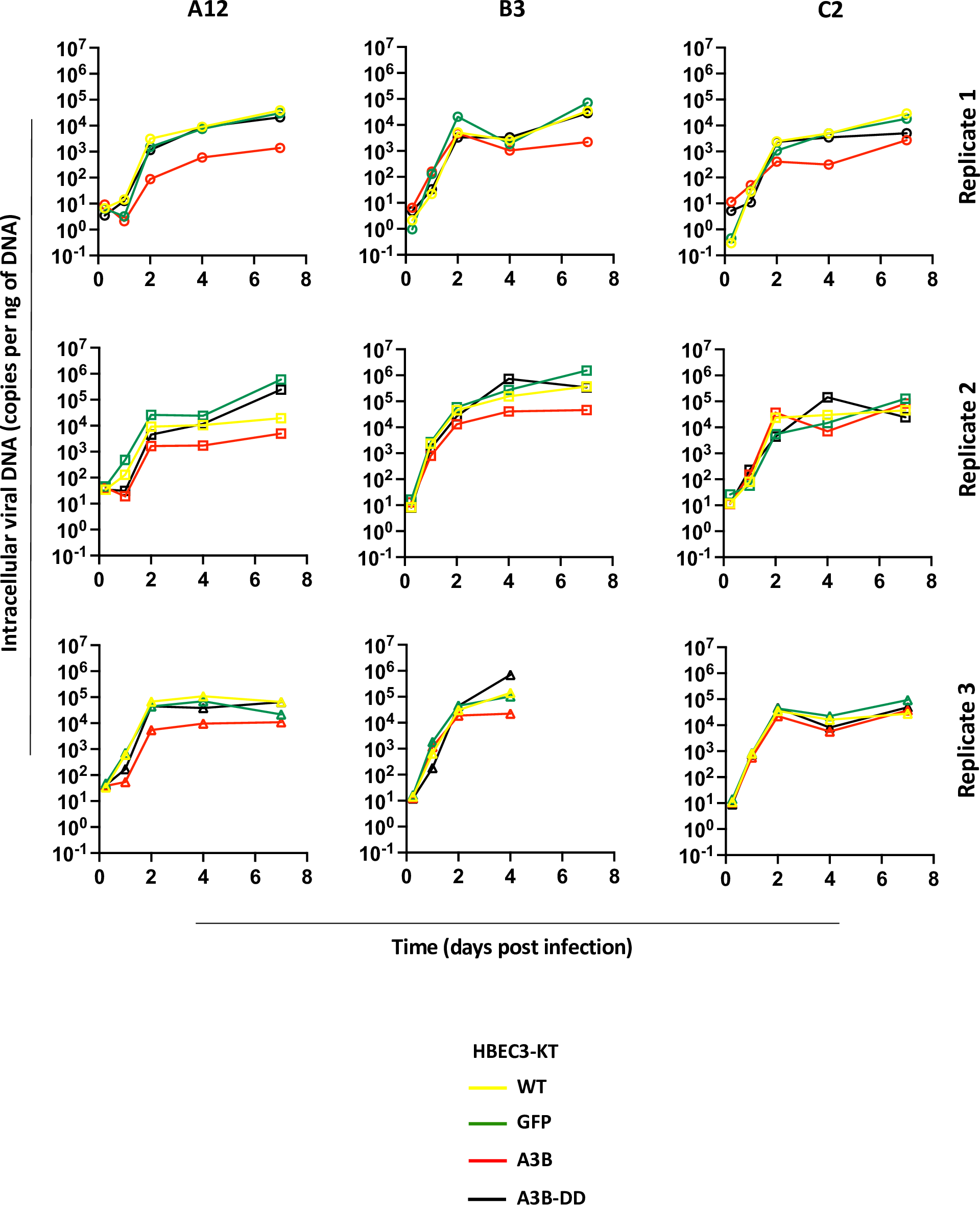

Supplement: S2 Fig — HBEC-WT, -GFP, -A3B and -A3B-DD were infected with HAdV-A12, -B3 or -C2 at a MOI = 0.03. Intracellular viral DNA levels were quantified by qPCR at 6- and 24-hours post infection and at 2-, 4- and 7-days post infection. The results for the three replicates are depicted. (TIFF) [file ppat.1011156.s002.tiff]

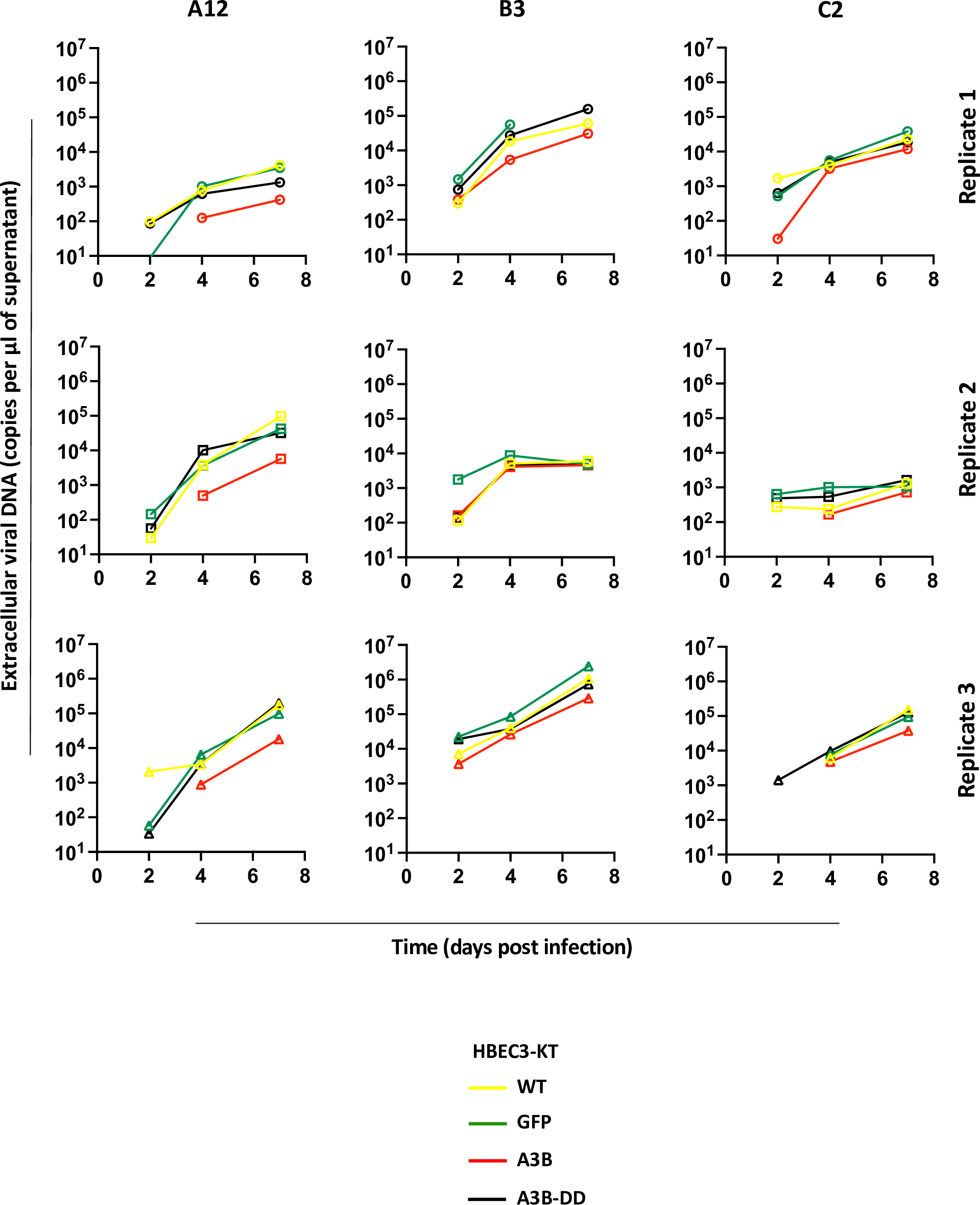

Supplement: S3 Fig — HBEC-WT, -GFP, -A3B and -A3B-DD were infected with HAdV-A12, -B3 or -C2 at a MOI = 0.03. Extracellular viral DNA levels were quantified by qPCR at 2-, 4- and 7-days post infection. The results for the three replicates are depicted. (TIFF) [file ppat.1011156.s003.tiff]

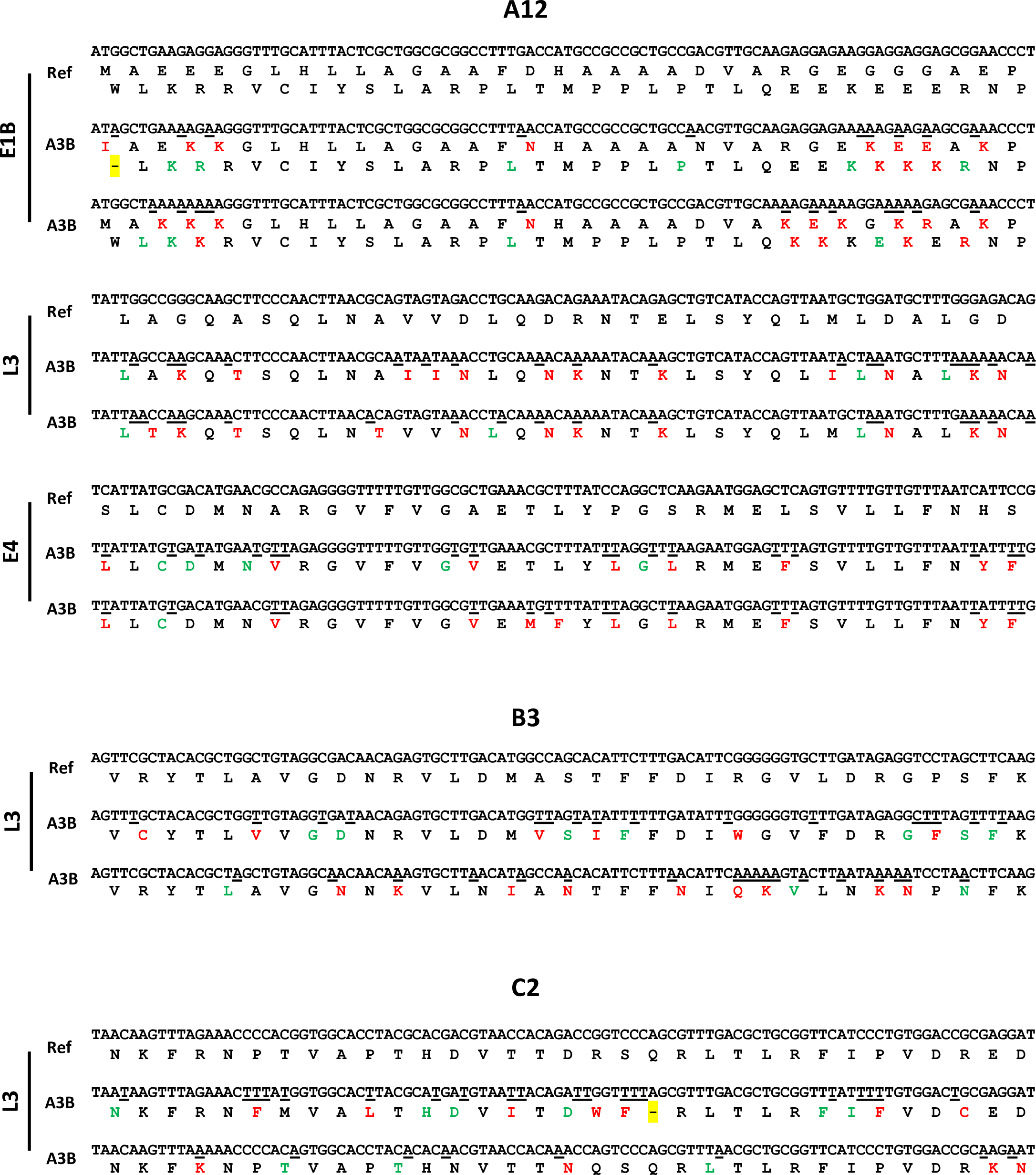

Supplement: S4 Fig — HBEC-WT, -GFP, -A3B and -A3B-DD were infected with HAdV-A12, -B3 or -C2 and the intracellular DNA was extracted 48 hours post infection. 3DPCR reactions conducted on different viral genes were cloned and sequenced. Hypermutated sequences detected in A3B-expressing cells (A3B) were aligned against the reference viral genome (Ref). The first 100 base pairs of representative examples are depicted. Mutated bases are underlined. Synonymous substitutions are colored in green, non-synonymous are colored in red and Stop codon are symbolized by a dash highlighted in yellow. (TIFF) [file ppat.1011156.s004.tiff]

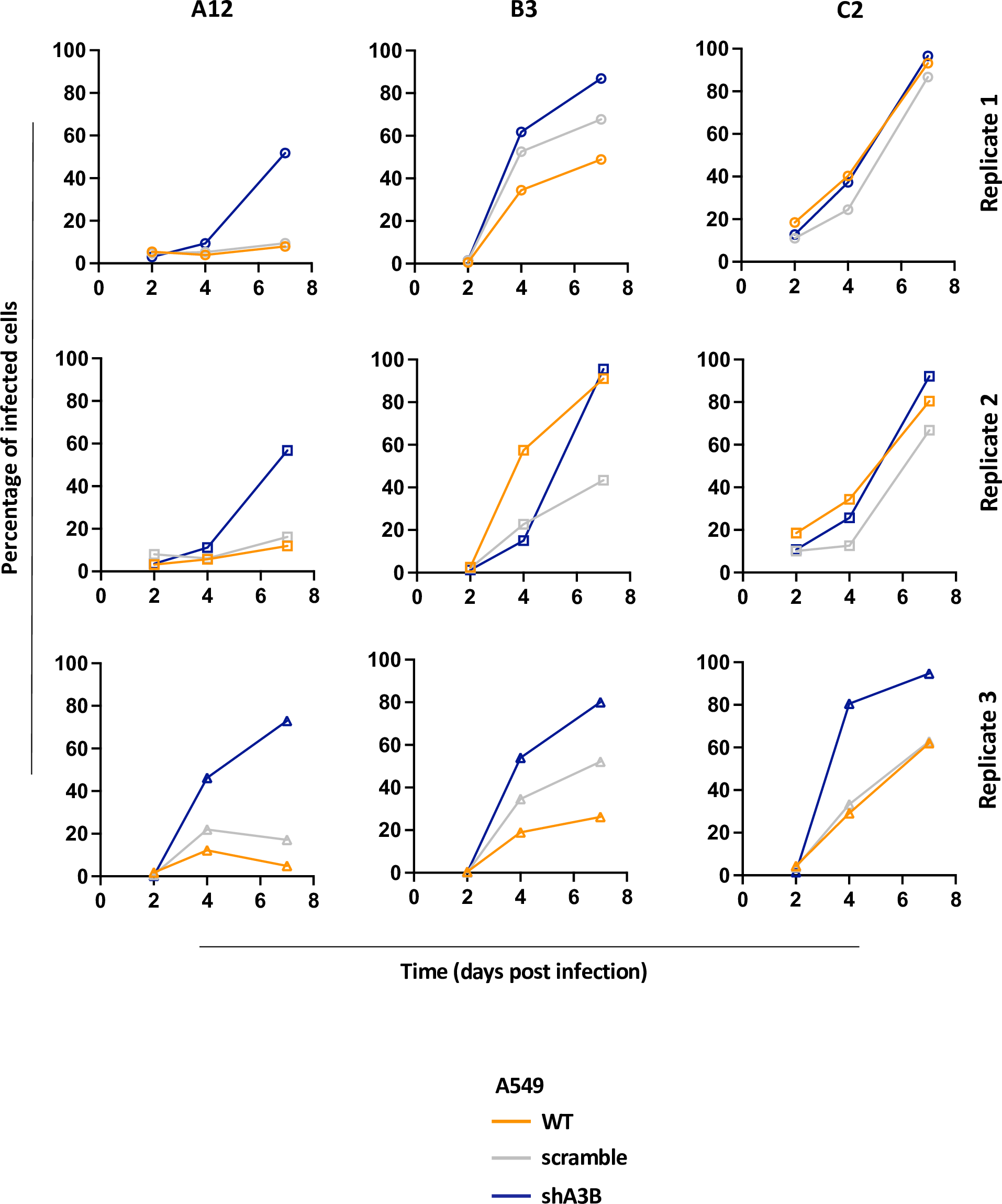

Supplement: S5 Fig — A549-WT, -scramble and -shA3B were infected with HAdV-A12, -B3 or -C2 at a MOI = 0.03. The percentage of infected cells were quantified by flow cytometry at 2-, 4-, 7-days post infection (dpi). The results for the three replicates are depicted. (TIFF) [file ppat.1011156.s005.tiff]

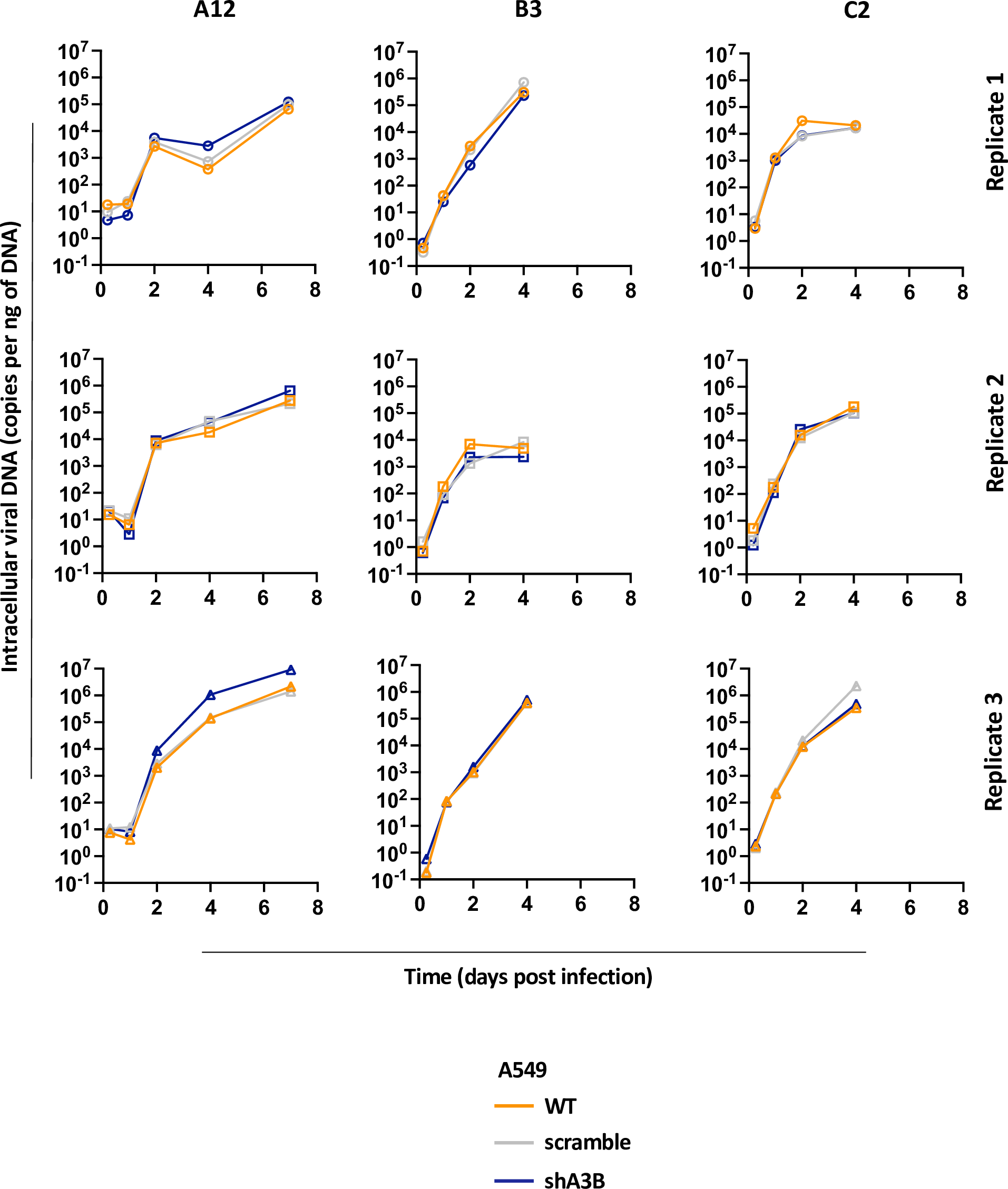

Supplement: S6 Fig — A549-WT, -scramble and -shA3B were infected with HAdV-A12, -B3 or -C2 at a MOI = 0.03. Intracellular viral DNA levels were quantified by qPCR at 6- and 24-hours post infection and at 2-, 4- and 7-days post infection. The results for the three replicates are depicted. (TIFF) [file ppat.1011156.s006.tiff]

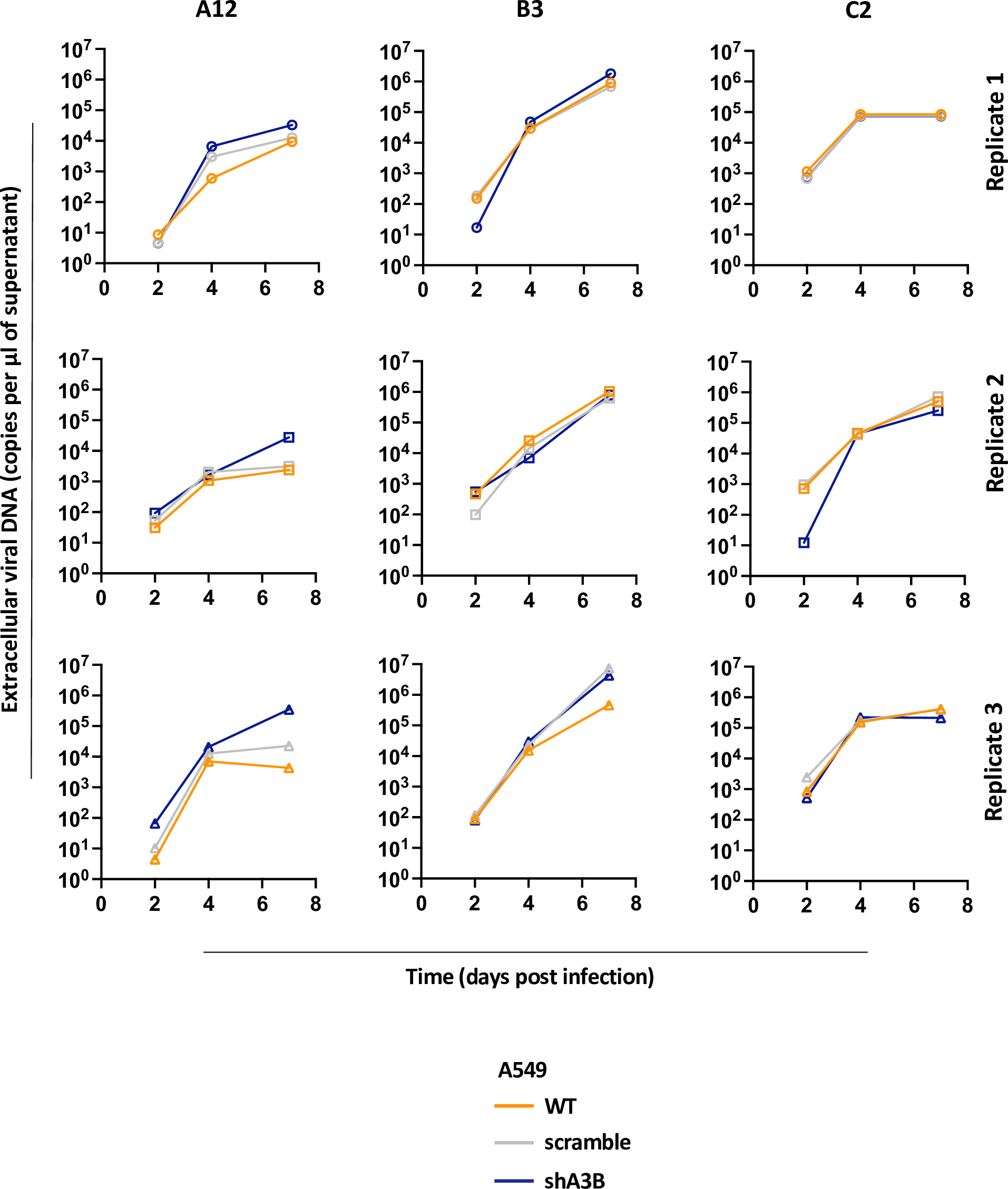

Supplement: S7 Fig — A549-WT, -scramble and -shA3B were infected with HAdV-A12, -B3 or -C2 at a MOI = 0.03. Extracellular viral DNA levels were quantified by qPCR at 2-, 4- and 7-days post infection. The results for the three replicates are depicted. (TIFF) [file ppat.1011156.s007.tiff]

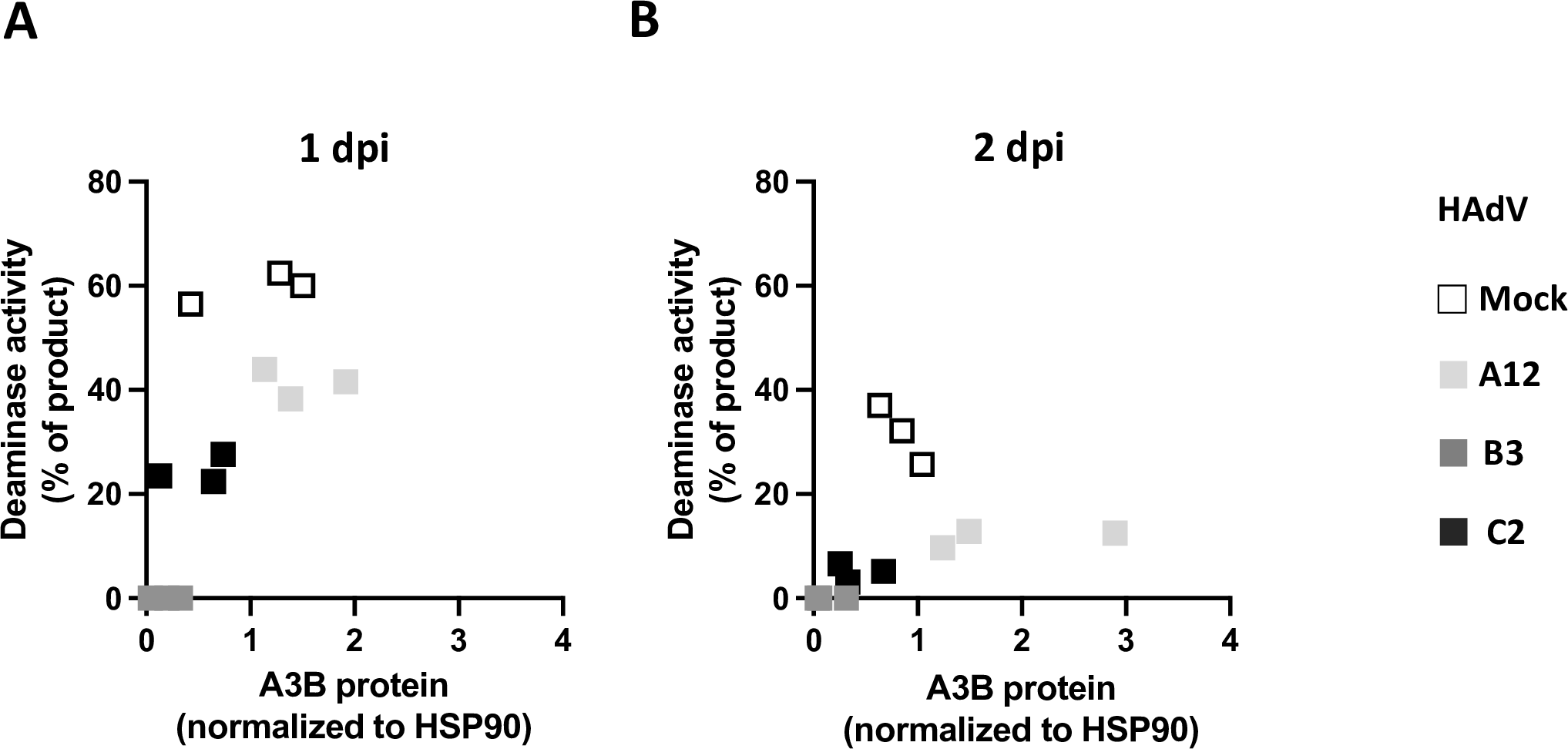

Supplement: S8 Fig — A549 cells were infected with HAdV-A12, -B3, -C2 or a mock control at MOI = 3. Cell extracts were performed after 1- and 2-days post-infection. A3B protein levels (X-axis) were plotted against cell lysate deaminase activities (Y-axis). (TIFF) [file ppat.1011156.s008.tiff]

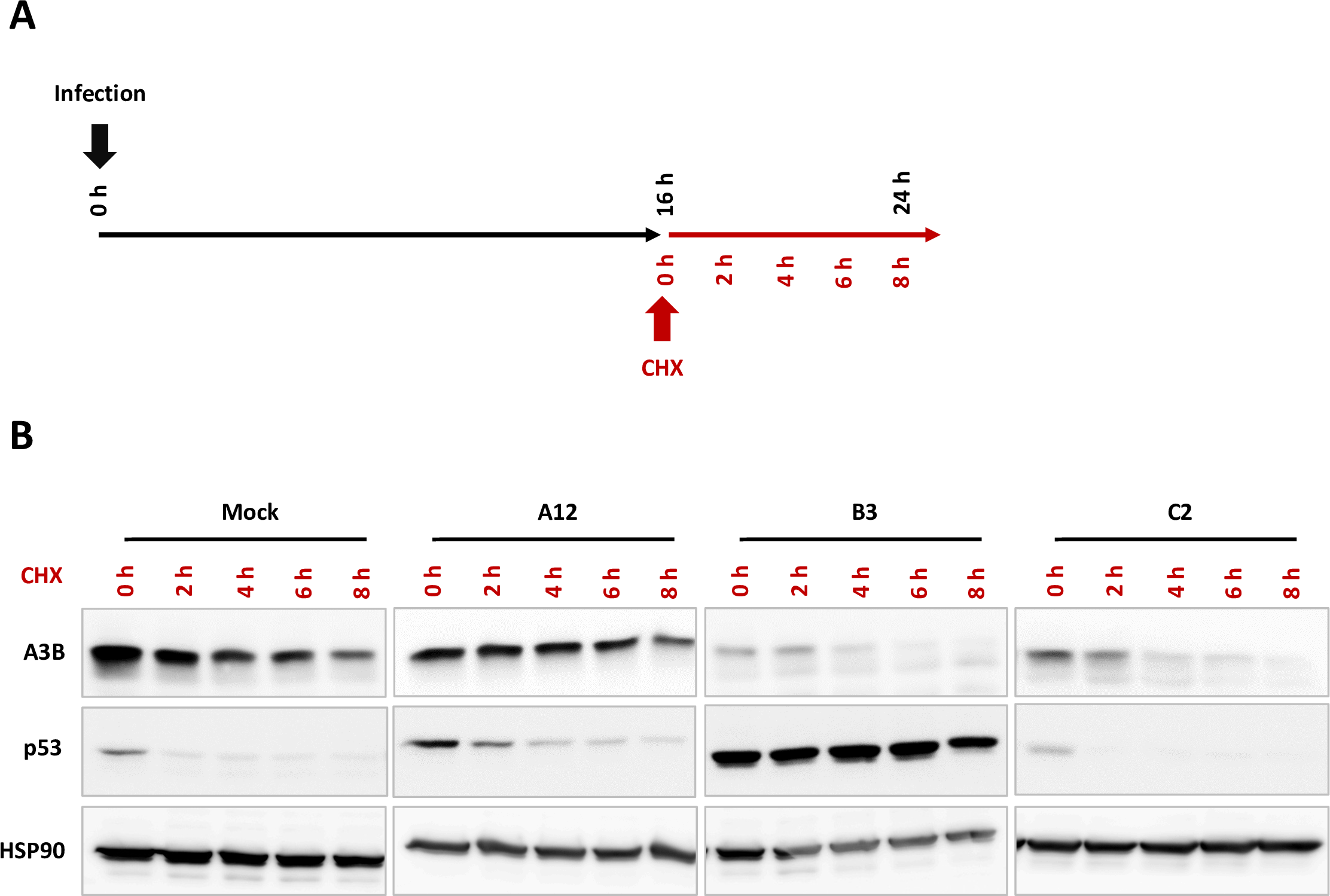

Supplement: S9 Fig — A549 cells were infected with HAdV-A12, -B3, -C2 or a mock control at MOI = 3. Cycloheximide was added at 16 hpi. The levels of A3B, p53 and HPS90 proteins were evaluated by western blot for a period of 8 hours. (TIFF) [file ppat.1011156.s009.tiff]

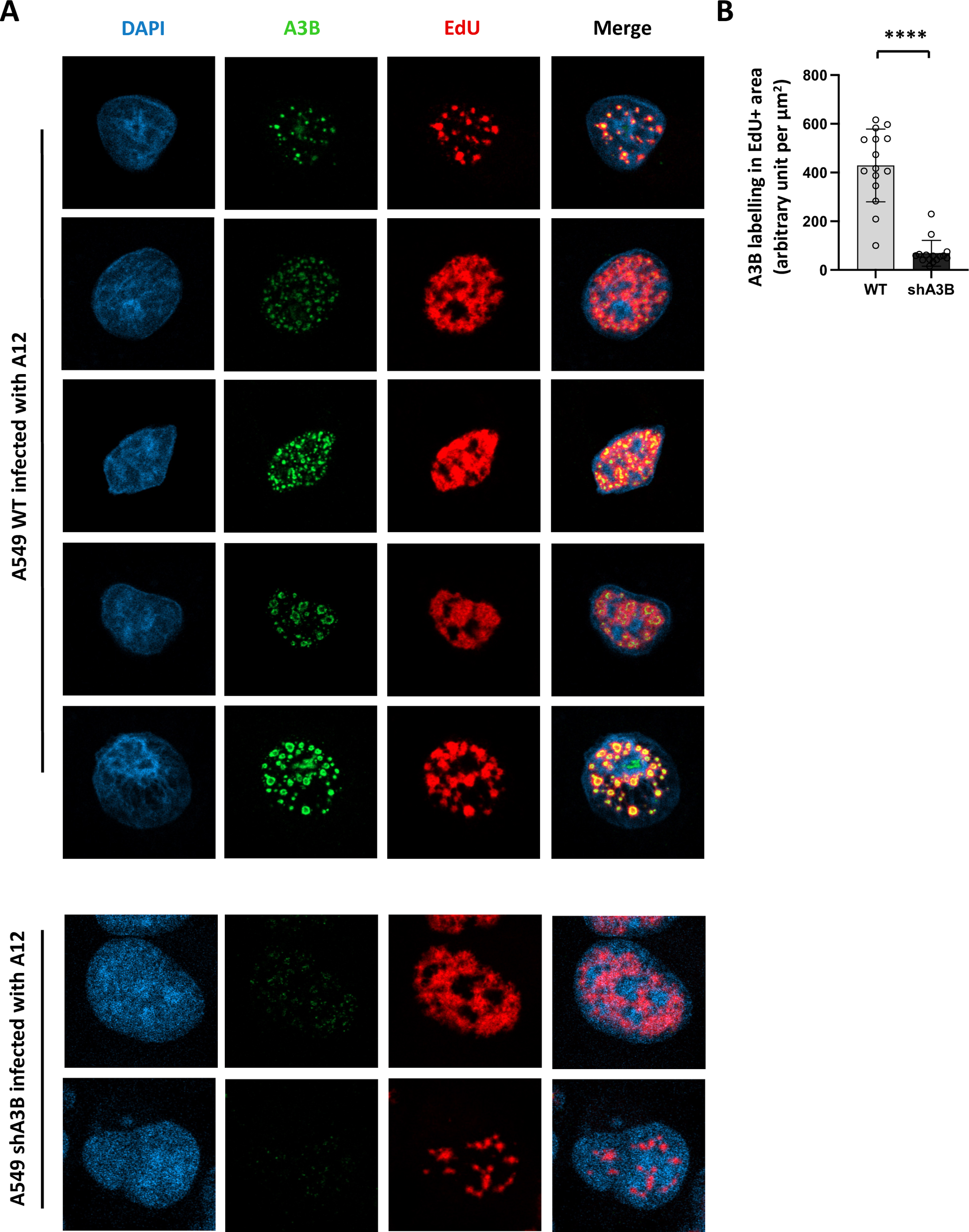

Supplement: S10 Fig — A549 WT or A549 shA3B were infected with HAdV-A12 at a MOI = 3 and analyzed 24 hours post infection. (A) The A3B protein (green) and the viral replication centers as identified by EdU labelling (red) were imaged by fluorescence microscopy. (B) A3B labelling was quantified within the EdU+ area. Each circle represents a given cell. P-values were calculated by unpaired t-tests. (TIFF) [file ppat.1011156.s010.tiff]
