# Supplementary material for: The APOBEC3B cytidine deaminase is an adenovirus restriction factor
Source: PLoS Pathog. 2023 Feb 6;19(2):e1011156. doi: 10.1371/journal.ppat.1011156 (PMC9934312; doi:10.1371/journal.ppat.1011156)
Supplement: S1 Text — Table A. Primers for cellular and viral mRNAs quantification. Table B. Primers for adenovirus DNA quantification. Table C. Primers and cycling conditions for 3DPCR. Table D. Primers for the discrimination of the APOBEC3B mRNA isoforms. (DOCX) [file ppat.1011156.s011.docx]

**Table A : Primers for cellular and viral mRNAs quantification**

| **Target** | **Application** | **Primer name** | **5’-3’ sequence** |
| --- | --- | --- | --- |
| APOBEC3B mRNA (isoforms 1 and 2) | mRNA quantification by RTqPCR | A3B_RTqPCR_Fw | GACCCTTTGGTCCTTCGAC |
|  | mRNA quantification by RTqPCR | A3B_RTqPCR_Rv | GCACAGCCCCAGGAGAAG |
| GAPDH mRNA | mRNA quantification by RTqPCR | GAPDH_RTqPCR_Fw | ATTCCCATCACCATCTTCCAG |
|  | mRNA quantification by RTqPCR | GAPDH_RTqPCR_Rv | CAGAGATGATGACCCTTTTGG |
| HAdV-A12  E1A mRNA | mRNA quantification by RTqPCR | E1A_A12_qPCR_Fw | TGAGCCTGAACCTAATAGCAC |
|  |  | E1A_A12_qPCR_Fw | CACAGCACATCTACGCCTC |
| HAdV-B3  E1A mRNA | mRNA quantification by RTqPCR | E1A_B3_qPCR_Fw | TGATGATGAGTCACCTTCTCCT |
|  |  | E1A_B3_qPCR_Rv | CCAGGCTTAGGCTTCACAG |
| HAdV-C2  E1A mRNA | mRNA quantification by RTqPCR | E1A_C2_qPCR_Fw | AACACACCTCCTGAGATACACC |
|  |  | E1A_C2_qPCR_Rv | AGGCTCGTTAAGCAAGTCC |
| HAdV-A12  E4orf6 mRNA | mRNA quantification by RTqPCR | E4orf6_A12_qPCR_Fw | TGCCGACTATGTTTTAGAGGG |
|  |  | E4orf6_A12_qPCR_Fw | TTGTAAAGACTTAAGTGAGATACGCA |
| HAdV-B3  E4orf6 mRNA | mRNA quantification by RTqPCR | E4orf6_B3_qPCR_Fw | TGGCAGAACAAGGAAGACCA |
|  |  | E4orf6_B3_qPCR_Rv | CCAATTACCACGATGTGAGGA |
| HAdV-C2  E4orf6 mRNA | mRNA quantification by RTqPCR | E4orf6_C2_qPCR_Fw | CCCTCATAAACACGCTGGAC |
|  |  | E4orf6_C2_qPCR_Rv | GCTGGTTTAGGATGGTGGTG |
| HAdV-A12  penton mRNA | mRNA quantification by RTqPCR | penton_A12-B3-C2_qPCR_Fw | ACCGTCAGTGAAAACGTTCC |
|  |  | penton_A12_qPCR_Fw | CAGTGCTTTGTAAACGTAGG |
| HAdV-B3 and HAdV C2  penton mRNA | mRNA quantification by RTqPCR | penton_A12-B3-C2_qPCR_Fw | ACCGTCAGTGAAAACGTTCC |
|  |  | penton_B3-C2_qPCR_Rv | CAGGGCCTTGTAAACGTAGG |

**Table B : Primers for adenovirus DNA quantification**

| **Target** | **Application** | **Primer name** | **5’-3’ sequence** |
| --- | --- | --- | --- |
| HAdV-A12 penton gene | vDNA quantification by qPCR | qPCR_HAdV-A12_penton_Fw | GCCCTTACAGATCACGGGAC |
|  |  | qPCR_HAdV-A12_penton_Rv | CAGTGCTTTGTAAACGTAGG |
| HAdV-B3 and -C2 penton gene | vDNA quantification by qPCR | qPCR_HAdV-B3+C2_penton_Fw | GCTCTCACAGATCACGGGAC |
|  |  | qPCR_HAdV-B3+C2_penton_Rv | CAGGGCCTTGTAAACGTAGG |

**Table C : Primers and cycling conditions for 3DPCR**

| **Target** | **Application** | **Primer name** | **5’-3’ sequence** | **Cycling conditions** |
| --- | --- | --- | --- | --- |
| HAdV-A12 E1A DNA | First round: classic PCR | A12 E1B 5 out | GTTTTCAGAGCGTTCGCCAGCTCTT | 95°C 5min, 40 x(95°C 30sec, 58°C 30sec, 72°C 2mn), 72°C 20mn |
|  |  | A12 E1B 3 out | CAGTTAGCCTACTAAGAACAGCAGCTT |  |
|  | Second round: 3DPCR | A12 E1B 5 in | TTCATCTGTGGGACGAACGGTTGCTT | 78-88°C 5min, 42 x(78-88°C 30sec, 60°C 30sec, 72°C 1min), 72°C 20mn |
|  |  | A12 E1B 3 in | CCTTCTTGCACCTGTTGTTCCATGTT |  |
| HAdV-A12 L3 DNA | First round: classic PCR | A12 L3 5 out | CAATCCAAGGATGACAACATTGAACTT | 95°C 5min, 40 x(95°C 30sec, 60°C 30sec, 72°C 2mn), 72°C 20mn |
|  |  | A12 L3 3 out | ATATTCCCAATGCCTATGTGGTTTGCTT |  |
|  | Second round: 3DPCR | A12 L3 5 in | AACCTTGAAATGCCAGACACGCATCTT | 75-83°C 5min, 42 x(75-83°C 30sec, 60°C 30sec, 72°C 1min), 72°C 20mn |
|  |  | A12 L3 3 in | GGAATTCCACAAGGAAAAGTACCGTGTT |  |
| HAdV-A12 E4 DNA | First round: classic PCR | A12 E4 5 out | AATGAACAAGCGACCTCTGAAACATGCTT | 95°C 5min, 40 x(95°C 30sec, 60°C 30sec, 72°C 2mn), 72°C 20mn |
|  |  | A12 E4 3 out | GTGTGGCTTTGGCGTGCTTGTAAGTT |  |
|  | Second round: 3DPCR | A12 E4 5 in | CAAACGGGTGGAAAAACAACTCTACTT | 75-83°C 5min, 42 x(75-83°C 30sec, 60°C 30sec, 72°C 1min), 72°C 20mn |
|  |  | A12 E4 3 in | GAGGGCGACTCTAATGCTTATGTGTT |  |
| HAdV-B3 E1A DNA | First round: classic PCR | B3 E1B 5 out | ACGGAGTCTCTGGCCTTTGGAGATT | 95°C 5min, 40 x(95°C 30sec, 60°C 30sec, 72°C 2mn), 72°C 20mn |
|  |  | B3 E1B 3 out | GGAGGGTCCAGGCCGCCTCTCGGATT |  |
|  | Second round: 3DPCR | B3 E1B 5 in | ATAGTCCGGGACTTTTTGAAGCTCTT | 76-82°C 5min, 42 x(76-82°C 30sec, 60°C 30sec, 72°C 1min), 72°C 20mn |
|  |  | B3 E1B 3 in | GGCTGCACTGGCCAGTAATCTAAGATT |  |
| HAdV-B3 L3 DNA | First round: classic PCR | B3 L3 5 out | CTTCAGTATGGGGAACAAATTTAGAA | 95°C 5min, 40 x(95°C 30sec, 56°C 30sec, 72°C 2mn), 72°C 20mn |
|  |  | B3 L3 3 out | AGTTTCCAAATTTACATTTTCCGTATAA |  |
|  | Second round: 3DPCR | B3 L3 5 in | CCGGGAAGACAATACCTACTCTTACAA | 80-86°C 5min, 42 x(80-86°C 30sec, 58°C 30sec, 72°C 1min), 72°C 20mn |
|  |  | B3 L3 3 in | CCCAATTTGCAAACCTTCTTTAGTAA |  |
| HAdV-B3 E4 DNA | First round: classic PCR | B3 E4 5 out | TCATAATGGAGTTGCTTCCTGACATT | 95°C 5min, 40 x(95°C 30sec, 60°C 30sec, 72°C 2mn), 72°C 20mn |
|  |  | B3 E4 3 out | ACCGTTGAGCACCTTGAAAATCGCATT |  |
|  | Second round: 3DPCR | B3 E4 5 in | GATAATTCAAGTACAGCCACACTCTT | 76-82°C 5min, 42 x(76-82°C 30sec, 55°C 30sec, 72°C 1min), 72°C 20mn |
|  |  | B3 E4 3 in | TGCCATCTGCGCGATCTACAATT |  |
| HAdV-C2 E1A DNA | First round: classic PCR | C2 E1B 5 out | GCGTAACTTGCTGGAACAGAGCTCTAA | 95°C 5min, 40 x(95°C 30sec, 60°C 30sec, 72°C 2mn), 72°C 20mn |
|  |  | C2 E1B 3 out | TTCCTAGCCTCCTCTGTAGCCTCAGAA |  |
|  | Second round: 3DPCR | C2 E1B 5 in | CTGTGGTGAGCTGTTTGATTCTTTGAA | 82-90°C 5min, 42 x(82-90°C 30sec, 60°C 30sec, 72°C 1min), 72°C 20mn |
|  |  | C2 E1B 3 in | GGTTAAAATGCGTCTCAGTTCTGGAA |  |
| HAdV-C2 L3 DNA | First round: classic PCR | C2 L3 5 out | CCCTGAAGCGCCGACGATGCTTYTAA | 95°C 5min, 40 x(95°C 30sec, 57°C 30sec, 72°C 2mn), 72°C 20mn |
|  |  | C2 L3 3 out | CCGCTTTTTGTAATTGTTTCTCCAGAYAA |  |
|  | Second round: 3DPCR | C2 L3 5 in | CGCCACCGAGACGTACTTCAGCYTRAA | 83-90°C 5min, 42 x(83-90°C 30sec, 60°C 30sec, 72°C 1min), 72°C 20mn |
|  |  | C2 L3 3 in | GTAGGCAGTGCCGGAGTAGRGYTTAA |  |
| HAdV-C2 E4 DNA | First round: classic PCR | C2 E4 5 out | GGCACAGCAGCGCACCCTGATCTCACTT | 95°C 5min, 40 x(95°C 30sec, 60°C 30sec, 72°C 2mn), 72°C 20mn |
|  |  | C2 E4 3 out | CGACGAGACCGGAGACGCAGATCTGTT |  |
|  | Second round: 3DPCR | C2 E4 5 in | CATAAACATTACCTCTTTTGGCATGTT | 82-90°C 5min, 42 x(82-90°C 30sec, 58°C 30sec, 72°C 1min), 72°C 20mn |
|  |  | C2 E4 3 in | GCACAACGTGAGTTACGTGCGAGGTCTT |  |

**Table D : Primers for the discrimination of the APOBEC3B mRNA isoforms**

| **Target** | **Application** | **Primer name** | **5’-3’ sequence** |
| --- | --- | --- | --- |
| A3B cDNA | Discriminations of the A3B transcriptional isoforms by RT-PCR | Ex4_F | ATGCCTTGGTACAAATTCGATG |
|  |  | Ex6_R | CCAAGTGACCCTGTAGATCTGG |
|  |  | Ex7_F | CTTTGTGTACCGCCAGGGAT |
|  |  | Ex8_R | TTTGCTGGTGTCTGTGAGCA |
