# Supplementary material for: The APOBEC3B cytidine deaminase is an adenovirus restriction factor
Source: PLoS Pathog. 2023 Feb 6;19(2):e1011156. doi: 10.1371/journal.ppat.1011156 (PMC9934312; doi:10.1371/journal.ppat.1011156)
Supplement: Attachment — Submitted filename: Response to Reviewers Lejeune_A3B_adeno_restriction.docx [file ppat.1011156.s012.docx]

**Resubmission for PPATHOGENS-D-22-01680: The APOBEC3B cytidine deaminase is an adenovirus restriction factor**

Dear Editors, Editor-In-Chief, dear Reviewers,

First of all, we thank you for considering our work and appreciate the time you spent reviewing our manuscript. Your requests and comments have greatly contributed to the improvement of the manuscript.

This letter contains a detailed list of our responses to review comments and a description of the changes we have made to the manuscript. Our detailed answers are colored in blue in the following text.

**Part I - Summary**

Please use this section to discuss strengths/weaknesses of study, novelty/significance, general execution and scholarship.

**Reviewer #1:** This manuscript by Lejeune and coworkers is the first to my knowledge to comprehensively assess the potential interaction between APOBEC3B (A3B) and adenoviruses. In general the work is well done, and I like the fact that most experiments involve 3 strains and biological/technical triplicates. The most important experiments show that A3B overexpression lowers AdV replication and increases the detection of hypermutants and A3B knockdown does the opposite. An intriguing aspect is that A-family AdV seem more susceptible than other families B and C also tested here. An unresolved question is how AdV protect themselves from completely lethal restriction. To date, all restriction-susceptible virus types have mechanisms to fight back (Vif for lentis, Bet for foamys, RNR for herpes, etc) but this can be a topic for future studies. My comments below are mostly to improve rigor and clarity.

**Reviewer #2:** The manuscript by Lejeune and colleagues provides evidence that the adenovirus genome is deaminated by APOBEC3B during virus replication. Their lab showed previously that adenovirus genomes bore signatures of APOBEC3 deamination and here they attempt to demonstrate this experimentally. Some of the data regarding the effects of over- and under-expression are not that convincing, in part because of the large variation between "biological" replicates. Nonetheless, it does appear that Ad12 may be more affected by APOBEC3B than are the B3 or C2 strains. The most interesting data in the manuscript is presented in Figure 7, where they show that APOBEC3B protein levels are decreased in cells infected with the B3 or C2 strains but not Ad12. If more data were presented about the mechanism by which this occurs, the significance and novelty of the manuscript would be greatly increased.

**Reviewer #3:** In this manuscript, Lejeune et al. found that A3B is a critical restriction factor suppressing adenovirus through a deaminase-dependent mechanism. The authors convincingly demonstrated that A3B prevents viral infection by directly inducing mutations in the virus genome leading to a decrease in viral replication and infectivity. Moreover, they found that certain adenovirus strains counteract A3B by inducing its degradation. The results presented in this study are very well executed without any major flaws.

We thank the reviewers for their constructive comments.

We have devoted most of our revision time to providing more insight into the mechanism(s) used by adenoviruses to antagonize A3B. Two new experiments were conducted and reported in Figure 7. A first experiment used the proteasome inhibitor epoxomicin to test whether A3B degradation was dependent of the proteasomal route (Fig 7E). A second experiment used the translation inhibitor cycloheximide to estimate the A3B protein half-life in infected and uninfected cells (Fig 7F). Interpretations of these experiments are detailed below.

**Part II – Major Issues: Key Experiments Required for Acceptance**

Please use this section to detail the key new experiments or modifications of existing experiments that should be absolutely required to validate study conclusions.

Generally, there should be no more than 3 such required experiments or major modifications for a "Major Revision" recommendation. If more than 3 experiments are necessary to validate the study conclusions, then you are encouraged to recommend "Reject".

**Reviewer #1: Major:**

1) Controls in Fig S1 should be merged into Fig 1. Readers need to understand the experimental system (maybe with cartoon) and the controls that were done to help establish it.

2) What is the A3B overexpression level in the cells in Fig 1, and how do these relate to physiological levels in primary cell types/tissues normally infected by adenoviruses? Such comparative data would be a nice supplementary figure.

3) The controls in Fig S5 for knockdown experiment are also very important and should be incorporated into the main Fig 5. As for #1 above, how do A3B levels in this cancer cell line compare to those observed in vivo in cell types/tissues normally infected by adenovirus?

4) The A3B westerns in Fig S5 and Fig7A raise the question of specificity. The band shown could be A3G, which sometimes migrates around 37kDa. A3B invariably migrates faster than 37kDa. Please show a control RTqPCR for related A3 family members, which will help demonstrate specificity (perhaps A3G is not expressed in this system) and that the observed changes are A3B-specific as implied here (which will also support Fig5/S5 studies). The 5210-87-13 mAb used for these experiments from the Harris lab recognizes a c-terminal epitope in A3A, A3B, and A3G so showing the full blot from 15- 50kDa will be additionally helpful for interpretation.

Reply for questions 1 to 4:

We moved the former Figure S1 panels into Figure 1 and the former Figure S5 panels into Figure 5 to better explain our experimental system and the controls made to validate it.

We introduced a new Figure S1 showing A3s expression in WT- and modified- A549 and HBEC3-KT cells. Figure S1 panel A shows the levels of A3B, A3C, A3F and A3G mRNAs in A549 or HBEC3-KT infected or mock-infected and in uninfected A549-shA3B and HBEC-A3B. The A3A, A3D and A3H transcripts were not detected. Figure S1 panel B shows a western blot from A549 and HBEC3-KT infected or mock-infected and from uninfected A549-shA3B and HBEC-A3B. We used the 5210-87-13 mAb and displayed the full blot from 15kDa up to 55kDa. Panel B notably shows how the A3B overexpression level (in HBEC-A3B) compares with levels observed in infected primary or cancer cells (A549 or HBEC3-KT). In A549 cells, A3G mRNA was below the quantification limit (Fig S1, panel A) and no band that could correspond to the A3G protein was observed by western blot (Fig S1, panel B). In HBEC3-KT, A3G mRNA was detected at a very low level (Fig S1, panel A) and a faint band can be seen at about 40kDa (Fig S1, panel B). In accordance with the mRNA data, no band corresponding to A3A could be observed under any of the conditions.

5) Mechanism of A3B counteraction by AdV unclear and may involve lowering A3B protein levels and/or deaminase activity. The authors begin to address this point in Fig 7. However, the experiments in Fig 7A and D should really be coupled --- ie. readers need to know both A3B protein levels and activity levels simultaneously, with appropriate controls. For instance, is the diminished activity in Fig 7D due to lower A3B protein levels or potentially to less extract in the deaminase activity reaction?

The Figure 7A and 7D are generated from the same protein extracts (in HED buffer for both the WB and deaminase tests). In Fig 7D, the same quantity of protein extract is loaded in each well (40 μg of protein per test, the details can be found in the material and method section). As suggested by the reviewer, we introduced a new Figure S8 showing the A3B protein levels and the deaminase activities for the different conditions at 1- and 2- dpi.

In the results section of the manuscript, we added the following text:

“Figure S8 displays A3B protein levels versus deaminase activity of cell extracts for mock-, A12-, B3-, or C2-infected cells, after 1 and 2 dpi and for all 3 replicates. It illustrates that the deaminase activity in cells infected with A12 is strongly reduced despite a still high amount of A3B protein.”

6) The colocalization experiment in Fig 8 is quite interesting. The authors should include their A3B KD/KO line as a specificity control (as well as RTqPCR data as requested above in comment #4).

We included pictures of A549 shA3B infected with the A12 strain in Figure S10. Quantification of the green signal (A3B) in the EdU+ area was reported in A549 WT and A549 shA3B (Fig S10B). We also included additional pictures of A549 WT infected with the A12 strain to further illustrate the A3B clusters in the nucleus.

In the results section of the manuscript, we added the following text:

“On the contrary, the A3B protein forms dense clusters in the nucleus of A12-infected cells (Figure 8A and Figure S10A for additional pictures). As labelling control, A549 shA3B cells were infected with the A12 strain. No A3B clusters could be observed in those cells (Figure S10).”

7) The replication center section of the discussion is confusing and misleading. The authors should scrap comparisons with cancer mutation (where no one has “caught” A3B in action) and focus instead on comparisons with other viruses. For instance, herpesviruses relocalize A3B (ref13,14) and PyV strangely induces A3B but the induced protein does not appear to co-localize with T-Ag/EdU positive replication centers (ref16). The latter paper has images that are comparable/contrastable to those here.

We agree that the comparisons with cancer mutation might be misleading/overreaching. We deleted the sentence: ” This is reminiscent of the editing activity of the A3s on tumor cells that mutate the cellular genome during DNA replication.”

We refocused the replication centre section of the discussion as follow:

“We observed that A3B restricts adenoviruses by a deaminase-dependent mechanism and showed that it colocalizes with the virus replication centres (as identified by their high level of EdU incorporation in A12-infected cells). This suggests that A3B edits adenoviruses during viral DNA replication. Of note, adenoviruses replicate by strand displacement. The displaced strand, being single-stranded, represents the prototypical substrate for A3B editing. It will be interesting to test whether A3B can be actively recruited to the displaced strand and whether it can replace some of the viral DNA-binding proteins (DBP) to act on the viral DNA.

Recruitment of A3B to virus replication centres in A12-infected cells is reminiscent of observations made in BK PyV-infected cells where A3B appears to access at least a subset of BK PyV replication centres [16]. On the other hand, it contrasts sharply with the active exclusion of A3B from replication centres of herpes viruses. Indeed, EBV and other herpes viruses like KSHV and HSV-1 encode viral proteins that relocate A3B to perinuclear bodies [13,14]. How adenovirus A12 and BK PyV cope with the presence of A3B in their genome replication centres remains an open question but low A3B editing activity might be beneficial for the genetic diversification of the virus (as discussed below).”

8) The authors suggest “...that adenoviruses evolved different mechanisms to antagonize APOBEC3B.” Alternatively, one mechanism might exist with different efficiencies depending on adenovirus species. Another might be that different species have different rates of aborted replication intermediates and what you’re detecting by 3DPCR may never be capable of replication. Please include preferred interpretation, as well as alternative interpretations, in discussion.

We discuss the possibility that the rate of aborted replicates varies between species (with a supposedly higher rate in A12-infected cells).

We added in the discussion section:

“In our tests, the three adenoviral strains were not equally sensitive to A3B, the replication kinetics of the B3 and C2 were much less impacted by A3B than that of A12. We did retrieve hypermutated viral sequences for the three strains, sequences that most likely will not lead to infectious viral particles. However, the 3DPCR does not allow their quantification. Thus, we speculate that the proportion of viruses carrying mutation clusters is higher during infection with the A12 compared to the other two strains. A formal investigation will require the set-up of a new technique based on high throughput sequencing.”

The data reported in Fig 7 and the new experiments reported in panel E (epoxomicin proteasome inhibitor) and panel F (CHX translational inhibitor) tend to show that the mechanism of antagonization of A3B differs between the species. In A12-infected cells, the A3B protein appears to be stabilized, yet partially inactive. In B3- and C2-infected cells, the A3B protein appears to be less expressed, perhaps through a translational inhibition. These results are further discussed in the revised manuscript.

**Reviewer #2:**

1. It is really not clear to me when during adenovirus replication the authors think APOBEC3B might be acting. APOBEC3 enzymes preferentially deaminate single-stranded DNA, but it was my impression that this doesn't really exist during adenovirus replication because the DNA BP coats the newly replicated ssDNA. Do the authors think that APOBEC3B displaces DBP?

We indeed consider this mechanism as a possibility. We added in the discussion section the sentence:

“Of note, adenoviruses replicate by strand displacement. The displaced strand, being single-stranded, represents the prototypical substrate for A3B editing. It will be interesting to test whether A3B can be actively recruited to the displaced strand and whether it can replace some of the viral DNA-binding proteins (DBP) to act on the viral DNA.”

2. Figure 3. Isn't 24hr post-infection too late to examine immediate early and early gene expression? Or does this represent virus spread? It seems to me that this expression should be examined earlier after infection. Also, do the authors have any explanation for as to why there is no difference in B3 penton transcripts, but there is a difference in infectious particle production?

Indeed, IE and E gene expression start between 8 to 12h post infection, rise and end up with a plateau after 24h to 30h. Late gene initiate transcription between 12 to 16h and rise steadily up to 36h to 48h. These timing are very much influenced by the viral strain and the cell type hosting the infection. (See Temporal dynamics of adenovirus 5 gene expression in normal human cells, Crisostomo et al., PLoS One, 2019).

We had actually performed the experiment after 12h and 24h. We initially chose to show only data at 24 hours because we though they were sufficient to deliver the message. We reckon that it might be better to also show the 12 hours data. Following reviewer #2 advices, we added the 12 hours data to Figure 3 and modified the text accordingly. We did not detect reliably penton mRNA expression at 12h. We did not observe any difference of E1A nor E4orf6 expression after 12h.

We do not know exactly why we did not detect a significant decrease of penton expression in B3 infected cells whereas we observed a significant decrease in term of viral DNA and infectious particles. We can however notice that average penton mRNA expression is lower in B3 infected cells than in mock even though the difference is not statistically relevant. Perhaps a higher number of replicates would lead to a significative difference.

3. Figures 4 and 6. The authors do report deamination of B3 and C2 genomes, both in over-expression and knockdown cells. Unfortunately, sequencing the products that amplify at lower temperatures doesn't allow one to look at differences in the extent of deamination between the different viruses, only the consensus sequences. It would be much more interesting to report whether the B3 and C2 genomes are less deaminated that Ad12, which would require a deep-sequencing approach. This would also support the data in Figure 7.

We agree that quantification of the extent of deamination and the comparison between the strains would bring valuable information. The design, set up and data interpretation of such deep-sequencing experiment would be very difficult to achieved in the 60-days resubmission window. We anticipate that the completion of such technic would need half a year to achieve but are considering it for future works. We chose to dedicate our time to better characterize the mechanism(s) employed by the different adenoviral strains to antagonize A3B (experiments using proteasome or translation inhibitors, Fig 7E and 7F).

4. Figure 7. Interestingly, B3 seems to reduce APOBEC3B levels more than C2, yet B3 replication was more affected than C2. How do the authors explain this? In part D), do the authors know if any other APOBEC3s are expressed in these cells?

In other words, is all the deaminase activity attributable to APOBEC3B? What happens to the activity in APOBEC3B knockdown cells?

It is true the B3 strain reduces the A3B protein level more efficiently than the C2 strain in A549 cells (Figure 7A). And, indeed, when the HBEC3-KT cells are synchronously infected (high MOI, Figure 3), the replication of the B3 strain is impacted by the overexpression of A3B whereas no change can be observed for the C2 strain. We cannot explain this phenomenon but can only speculate. We observed that the A12 strain induces a decrease of A3B enzymatic activity. Perhaps the C2 strain is also able to inhibit A3B activity and this inhibition could intervein more rapidly than the decrease of the A3B protein amount.

We added a new supplemental figure (Figure S1) that displays the levels of A3B, A3C and A3F mRNAs in the A549 cells (mock or infected). A3A, A3D, A3G and A3H mRNA levels were below the quantification limit. The deaminase activity measured in A549 cells seems to be entirely attributable to A3B as nearly no deamination could be observed in A549 A3B KD (Figure 5 panel C, third lane).

**Reviewer #3:** My main criticism is there is no data supporting that the decrease of A3B protein level is mediated through a degradation process after B3 and C2 infection. Can the level of A3B be restored by blocking the proteasome, certain viral proteins, or specific ubiquitin ligase complexes?

The use of the proteasome inhibitor epoxomicin did restore the some of the A3B protein level in C2- or B3-infected conditions (Figure 7E). Importantly, epoxomicin treatment also led to an increase of A3B protein in mock-infected cells. These observations are in line with the work of Scholtés et al. demonstrating that A3B protein amount is regulated by proteasomal degradation.

Using cycloheximide, we observed that the A3B protein half-life was similar in mock-, B3- and C2-infection conditions but was increased by infection with the A12 strain (Figure 7F).

Overall, these data show that A3B protein is degraded by the proteasomal route in B3- and C2-infected cells, the kinetics of degradation being similar to the kinetics observed in mock-infected cells. This suggests that the decreased amount of A3B in B3- and C2-infected cells is rather due to an inhibition of A3B mRNA translation.

On the contrary, the A3B protein is stabilized in A12-infected conditions.

These results are discussed in detail in the revised manuscript.

**Part III – Minor Issues: Editorial and Data Presentation Modifications**

Please use this section for editorial suggestions as well as relatively minor modifications of existing data that would enhance clarity.

**Reviewer #1: Minor:**

9) Are most experiments here true biological replicates (different virus stocks/cells on different days), or a technical replicates from replica experiments run in parallel at same time with the same viral stocks/cells?

We clarified the notion of ‘biological replicate’ by stating:

"The experiments were conducted in three biological replicates, with each replicate being independent (i.e. cells plated and infected on different days)." Also, we used the same viral stocks between replicates.

10) Why do the authors rely on a single A3B knockdown construct instead of a much cleaner crispr knockout, at least for further validation?

We used shRNAs to knock-down A3B rather than Crispr editing for technical reasons and cost-issues.

We had in our hands 3 lentiviral vectors delivering shRNAs against the A3B mRNA. These constructs already proved their efficiency and specificity.

For the establishment of A3B KO clones by Crispr editing, we would first need to transduce the cells with a lentivirus delivering the Cas9, then transduce with a lentivirus delivering the sgRNA and select with antibiotics or FACS-sorting. After clone selection, we would finally need to remove the integrated Cas9 gene as we are not comfortable with studying the replication of the adenoviral genome in cells over-expressing an endonuclease in the nucleus (we do not know whether the Cas9 can interfere with the adenovirus genome replication but did not want to take the risk). The construct of an A3B KO cell line by Crispr would require more steps, take longer and would be more costly (money is a constraint for the lab).

11) Mutation clusters should be called exactly that – strand-coordinated clusters (or kataegis); hypermutation/editing is more general, and as the authors are aware originally associated with hypermutated HIV-1 genomes (high almost everywhere and not necessarily clustered). Clustered changes are, by definition, a requirement for 3DPCR, as the primer pairs only sample very short DNA regions and lower-denaturation temp amplicons only appear if multiple G/C-to-A/T changes have lowered the overall H-bond potential...

12) That said, can anything be inferred based on the strand that is accumulating C-to-U changes, as all of the reported clusters appear to be strand-biased...? Is this the same strand of the AdV genome?

Reply for questions 11 and 12:

We modified our text several times to specifically describe the mutations introduced by A3B as “strand-coordinated C to T clustered mutations” or “mutation clusters”.

It is true that all sequences obtained carry strand-coordinated mutations (i.e., either C to T or G to A). We have never observed a sequence with both C to T and G to A clustered mutations. However, we have observed mutation clusters on both strands of the same gene (from different 3DPCR amplicons). Figure S4 illustrates representative sequences carrying clustered C to T or G to A mutations. For instance, for the L3 gene, we found 3DPCR amplicons with C to T mutations and other amplicons with G to A mutations.

We have added this information to the revised manuscript.

**Reviewer #2:**

1. Line 93. The TC motif is not favored by APOBEC3G. Although the authors mention this later on, it should be stated here.

Indeed, we have modified the sentence as follows:

“We demonstrated that adenovirus genomes were depleted for the 5’TC motif that is favored by most of the A3s [21].”

2. I was unclear as to what the authors meant throughout the manuscript by "biological replicates". Were these technical replicates or experimental replicates? If the former, then experimental replicates are needed to do statistics.

We clarified the notion of ‘biological replicate’: “The experiments were conducted in three biological replicates, each replicate being independent (i.e. cells plated and infected on different days).” Also, we used the same viral stocks between replicates.

**Reviewer #3:**

- What is the expression level of the other APOBEC3 family members in A549 and HBEC cells? Could other APOBEC3s expressed in these cell lines also participate in adenovirus restriction?

We added a new Figure S1 showing the expression of the different A3s in WT- and modified- A549 and HBEC3-KT cells. Figure S1 panel A shows the levels of A3B, A3C, A3F and A3G mRNAs in infected or mock-infected A549 and HBEC3-KT and in uninfected A549-shA3B and HBEC-A3B. The A3A, A3D and A3H transcripts were not detected. Figure S1 panel B shows the A3B protein levels in those cells.

HBEC3-KT cells infected with adenoviruses express A3B, A3C, A3F and A3G (albeit to a very low level) (Figure S1 and Lejeune et al., JVI, 2020). No expression of A3A, A3D and A3H was detected. We cannot completely rule out the role of A3C, A3F or A3G in adenovirus restriction but the KD of A3B in HBEC3-KT cells completely abrogates the deaminase activity of the protein extract (Lejeune et al., JVI, 2020).

A549 cells infected with adenoviruses express A3B, A3C and A3F (Figure S1). No expression of A3A, A3D, A3G and A3H was detected. Once again, we cannot completely rule out the role of A3C or A3F in adenovirus restriction but the KD of A3B in A549 cells completely abrogates the deaminase activity of the protein extract (Figure 5C).

- The deaminase assay presented in Fig7D and S5C have no negative control without cell extract. In addition, the difference in DNA deaminase activity between the mock and A12 could be better illustrated, for example with cell extract titration.

The probe incubated without cell extract migrates at the same level as the upper band shown in our gels. We illustrated the difference in deaminase activity between mock and A12 by another supplemental figure. Because figures 7A and 7D are generated from the same protein extracts (in HED buffer for both the WB and deaminase tests), we introduced a new Figure S7 showing the A3B protein levels and the deaminase activities for the different conditions at 1- and 2- dpi. Importantly, in Fig 7D, the same quantity of protein extract is loaded in each well (40 ug of protein per test, the details can be found in the material and method section).

In the results section of the manuscript, we added the following text:

“Figure S8 displays A3B protein levels versus deaminase activity of cell extracts for mock-, A12-, B3-, or C2-infected cells, after 1 and 2 dpi and for all 3 replicates. It illustrates that the deaminase activity in cells infected with A12 is strongly reduced despite a still high amount of A3B protein.”

-Is the localization of A3B to the viral replication center mediated by the NTD or CTD domains that are both known to bind single-stranded DNA?

This is also a very interesting question, to which we have not the answer yet. We are conducting several experiments at the moment to: i. identify the possible viral protein implicated in the A3B antagonization and ii. identify the mechanisms allowing A3B to access the viral replication centers (at least in A12 infected cells). We are constructing several versions of A3B to tackle this question.

PLOS authors have the option to publish the peer review history of their article (what does this mean?). If published, this will include your full peer review and any attached files.

If you choose “no”, your identity will remain anonymous but your review may still be made public.

Do you want your identity to be public for this peer review? For information about this choice, including consent

withdrawal, please see our Privacy Policy.

Reviewer #1: No

Reviewer #2: No

Reviewer #3: Yes: Rémi Buisson
