# Supplementary material for: The APOBEC3B cytidine deaminase is an adenovirus restriction factor
Source: PLoS Pathog. 2023 Feb 6;19(2):e1011156. doi: 10.1371/journal.ppat.1011156 (PMC9934312; doi:10.1371/journal.ppat.1011156)
Supplement: Attachment — Submitted filename: 2023_01_22_Response to reviewers.docx [file ppat.1011156.s013.docx]

**Resubmission for PPATHOGENS-D-22-01680R1: The APOBEC3B cytidine deaminase is an adenovirus restriction factor**

Dear Editors, Editor-In-Chief, dear Reviewers,

First of all, we thank you for considering our work and appreciate the time you spent reviewing our manuscript. Your requests and comments have greatly contributed to the improvement of the manuscript.

This letter contains a detailed list of our responses to review comments and a description of the changes we have made to the manuscript. Our detailed answers are colored in blue in the following text.

Reviewer's Responses to Questions

Part I - Summary

Please use this section to discuss strengths/weaknesses of study, novelty/significance, general execution and scholarship.

Reviewer #1: The authors have done a nice job with revisions and have addressed all of my comments.

Reviewer #2: The authors have provided some additional data to support their observations that A12 but not B3 or C2 are affected by A3B, with the latter 2 viruses contributing to A3B degradation by an unknown mechanism. There are still many unanswered questions regarding the significance of their findings in the context of adenovirus replication.

Reviewer #3: The authors have addressed all my comments.

Part II – Major Issues: Key Experiments Required for Acceptance

Please use this section to detail the key new experiments or modifications of existing experiments that should be absolutely required to validate study conclusions.

Generally, there should be no more than 3 such required experiments or major modifications for a "Major Revision" recommendation. If more than 3 experiments are necessary to validate the study conclusions, then you are encouraged to recommend "Reject".

Reviewer #1: None

Reviewer #2: 1. When A3B would have the opportunity to deaminate DNA. To me, this is a critical question. The authors explanation would suggest that that A3B binds more tightly to ssDNA than does the DNA BP. This doesn’t seem likely to me.

In our previous paper, we observed an APOBEC3 footprint located at the ends of the adenoviral genome and specifically on the displaced strands (Poulain et al., Plos Pathogens, https:// doi.org/10.1371/journal.ppat.1008718). We hypothesize that A3B is actively recruited to the displaced strand and replaces some of the viral DNA-binding proteins to act on the viral DNA. Still, this remains to be formally demonstrated (or disproved).

2. Timing of effect. There is no difference between in IE or E gene expression at 12 hr post-infection, and the only difference was at 24 hr in E4orf6 and penton expression with A12. I’m not sure how to reconcile these results – if the effect is only seen after virus spread, there should also be differences E1A expression at 24 hr. Or is this because there are fewer GC pairs in the E1A gene, so it is less susceptible to deamination? Have the authors looked at deamination in regions of the Ad genome besides E4? I still think there is a lack of correlation between different sets of data (penton expression in B3 infected cells is not reduced, but infectious particles are reduced).

We recognize that these data are difficult to interpret and provide here our personal interpretation.

A3B slows down the progression through the E and L phases. By doing so, less E and L transcripts are being produced and therefore less virions. A3B does not seem to block viral transcription directly as IE mRNA levels are not impacted. A3B does not seem to impair viral DNA polymerase processivity as the A3B-DD protein does not restrict adenoviral propagation.

The E1 genes are indeed depleted for 5’TC motifs (Poulain et al., Plos Pathogens, https:// doi.org/10.1371/journal.ppat.1008718, see supplemental figures S8 and S9), and are therefore less susceptible to deamination. However, this is also the case for the E4 genes.

We have looked for deamination in the E1B, L3 and E4 regions. We chose these regions as there are located at different parts of the viral genome (respectively at the ‘right’ end, in the middle and at the “left” end of the viral genome). Deamination can be observed in these 3 regions.

We were also puzzled to see a reduction of virion production in B3-infected and A3B expressing cells whereas no significant change was observed for penton and E4 expression. Deamination of the viral genome may not reduce viral transcription but nevertheless strongly impacts the production of functional viral proteins and therefore the assembly and release of infectious viral particles.

3. 3. The authors now suggest that B3 and C2 at least partially degrade A3B. I still feel that this part of the manuscript is the most interesting and novel yet is under-developed – is there a viral protein that is responsible for this? I’m still not clear as to what the authors think is happening with Ad12 (“deaminase activity in cells infected with A12 is strongly reduced despite a still high amount of A3B protein.”), given that A3B does affect its infection.

Our data show that the A3B protein level is decreased by B3 and C2 infection. We are indeed hypothesizing that one or more viral proteins can promote the degradation of A3B and are trying to identify them.

Regarding A12, we believe that the virus is not able to reduce the amount of A3B protein but can, at least partially, inhibit its enzymatic activity. We are currently trying to identify the molecular players involved.

Reviewer #3: It would be important to mention that epoxomicin treatment does not fully restore A3B level after B3 infection suggesting that it may have an additional mechanism leading to the decrease of A3B protein level

We agree with the reviewer and have added the following sentence to the manuscript:

“It is important to note that epoxomicin treatment does not fully restore A3B level after B3 infection suggesting that it may have an additional mechanism leading to the decrease of A3B protein level.”

Part III – Minor Issues: Editorial and Data Presentation Modifications

Please use this section for editorial suggestions as well as relatively minor modifications of existing data that would enhance clarity.

Reviewer #1: None

Reviewer #2: (No Response)

Reviewer #3: (No Response)
